# Knowledge, Attitude, and Practice Regarding Water Intake Among University Students in China’s Seven Geographical Divisions: A Cross-Sectional Analysis

**DOI:** 10.3390/nu18020225

**Published:** 2026-01-10

**Authors:** Haiyue Yang, Jianfen Zhang, Shuyi Zhou, Yongye Song, Yi Zhang, Yunxia Zhu, Na Zhang

**Affiliations:** 1 Department of Nutrition and Food Hygiene, School of Public Health, Peking University, 38 Xue Yuan Road, Haidian District, Beijing 100191, China; 2411210094@stu.pku.edu.cn (H.Y.); zjf@bjmu.edu.cn (J.Z.); 2211110217@stu.pku.edu.cn (S.Z.); songyongye@bjmu.edu.cn (Y.S.); 2010108611@stu.pku.edu.cn (Y.Z.); 1810306128@pku.edu.cn (Y.Z.); 2 National Institute for Nutrition and Health, Chinese Center for Disease Control and Prevention, 27 Nanwei Road, Xichen District, Beijing 100050, China; 3 Laboratory of Toxicological Research and Risk Assessment for Food Safety, Peking University, 38 Xue Yuan Road, Haidian District, Beijing 100191, China

**Keywords:** water intake, knowledge-attitude-practice (KAP), health education, geographical divisions

## Abstract

**Background**: Inadequate water intake is prevalent among Chinese college students, a group at a critical stage for establishing lifelong health habits. However, nationwide data on their knowledge, attitudes, and practices (KAP) regarding water intake remain scarce. This study aims to describe regional variations in water-related KAP among undergraduates across seven major geographical regions of China, providing evidence for developing targeted health promotion strategies. **Methods**: A cross-sectional survey employed multistage stratified convenience sampling to recruit undergraduate students (N = 3161) from one university in each of China’s seven regions. Participants completed a KAP questionnaire. Data analysis utilized chi-square tests with Bonferroni correction, reporting effect sizes and confidence intervals. **Results**: A total of 3161 valid responses were obtained (response rate: 98.3%). Students in South China demonstrated the lowest awareness of regular water intake (52.0%) but the highest awareness of daily recommended water intake (32.9%). South China and Northeast China exhibited weaker recognition of water’s importance (65.6% and 94.0%, respectively) and the lowest prevalence of “thirst-driven” drinking behavior (21.7% and 32.4%, respectively). **Conclusions**: The knowledge, attitude, and practice (KAP) status regarding water consumption among Chinese university students is concerning and exhibits significant regional disparities. Key issues include knowledge gaps, disconnect between attitudes and behaviors, and deeply ingrained unscientific drinking habits. Analysis based on KAP theory indicates that future health promotion strategies must move beyond mere knowledge dissemination and adopt region-specific, multilevel comprehensive interventions.

## 1. Introduction

Water is one of the essential substances for human survival and development. It participates in the body’s metabolic processes, helps maintain the normal osmotic pressure of body fluids, balances electrolytes, and regulates body temperature [1]. Inadequate water intake may impair cognitive function [2,3,4] and physical performance [5,6], and it can also increase the incidence and prevalence of kidney diseases and urinary stones [7,8,9]. According to the *Chinese Dietary Reference Intakes (2023)*, the recommended daily water intake is 1700 mL for male adults and 1500 mL for female adults [10].

College students are in the early stages of adulthood, a critical period for physical and mental development and the formation of healthy behaviors. During this period, higher cognitive functions in the brain continue to develop [11], and the lifestyle habits established now profoundly influence long-term health trajectories. However, studies indicate that Chinese college students commonly suffer from inadequate water intake, with high rates of dehydration and low proportions achieving optimal hydration levels [12,13,14,15,16]. Therefore, investigating and improving this group’s drinking behavior is critically important.

This study employs the knowledge–attitude–practice (KAP) theory as its core theoretical framework. This model serves as a fundamental tool widely applied in public health research. Its clear structure enables systematic descriptive diagnosis of specific populations’ health knowledge, attitudes, and behaviors, making it particularly suitable for establishing baseline data and identifying key problem areas [17,18]. This aligns with the preliminary survey objective of comprehensively mapping the current status of knowledge, attitudes, and practices regarding drinking water among Chinese college students and identifying regional disparities. The model deconstructs the formation and modification of health behaviors into a linear progression of “knowledge → attitude → behavior,” positing that knowledge serves as the foundation, attitude as the motivator, and behavior as the target [19,20]. This framework exhibits unique applicability to drinking behavior research: water consumption is a fundamental daily health behavior significantly influenced by awareness, where deficiencies often stem from knowledge gaps (e.g., unaware of recommended intake), attitudinal biases (e.g., believing thirst is the sole indicator for drinking), or poor behavioral habits (e.g., substituting plain water with sugary beverages). The KAP model’s clear structure enables systematic evaluation of these three critical components. We acknowledge its limitation, however, that the transition from knowledge to attitude to behavior is not strictly linear. Future research may integrate frameworks like the Theory of Planned Behavior to further explore the underlying psychological and social drivers of behavioral change [18].

Previous studies on university students’ drinking habits have preliminarily applied the KAP model [21]. For instance, research has examined differences in knowledge, attitude, and practice among students of varying genders, majors, and academic years, revealing higher knowledge awareness among female students, medical students, and upperclassmen [22,23]. However, these studies have theoretical and methodological limitations: Methodologically, previous surveys were often confined to single institutions, single cities, or specific seasons, resulting in limited sample representativeness and generalizability of conclusions. Compared to previous studies, this research not only theoretically focuses on describing the current state of knowledge, attitude, and practice but also incorporates geographical region as a core macrolevel influencing factor into the analytical framework. This aims to examine whether and how each dimension of KAP is affected by regional environment and culture. Methodologically, this study pioneers a nationwide sampling survey across seven major geographical regions to obtain more nationally representative data, addressing the geographical coverage limitations of prior research.

This study represents the first nationwide investigation systematically examining differences and characteristics in college students’ knowledge, attitudes, and practices (KAP) regarding water intake from a geographic regionalization perspective. China’s vast territory encompasses seven major geographic regions (e.g., North China, East China, South China, Central China, Southwest China, Northwest China, Northeast China), which exhibit significant variations in climatic conditions (e.g., humidity, temperature), water resource abundance, dietary cultures, and living customs [24,25]. Macro-environmental and cultural factors likely subtly shape individuals’ drinking water knowledge and behavioral habits. However, existing research has entirely overlooked this crucial macrolevel variable, focusing solely on individual or microgroup characteristics (e.g., gender, major). Identifying and validating differences arising from geographic regions not only deepens our understanding of the root causes of variations in drinking behavior but also provides essential scientific evidence for developing region-specific health education strategies in the future. This, in turn, enhances the targeting and effectiveness of interventions. Therefore, filling this research gap holds significant theoretical value and practical implications.

## 2. Materials and Methods

### 2.1. Research Design and Ethics

This study is a nationwide cross-sectional questionnaire survey conducted simultaneously between May and June 2023. The research protocol was approved by the Peking University Biomedical Ethics Committee (Ethics Review Board approval number: IRB00001052022119), and all participants signed informed consent forms prior to the survey.

### 2.2. Sampling and Subjects

A multistage stratified simple sampling method was employed. First, stratification was conducted based on China’s seven major geographical regions (East China, South China, North China, Central China, Southwest China, Northwest China, and Northeast China). To ensure selected cities reflect typical climatic, cultural, and economic development characteristics of their regions, one representative city was conveniently sampled from each zone: Shanghai (East China), Haikou (South China), Tianjin (North China), Changsha (Central China), Kunming (Southwest China), Lanzhou (Northwest China), and Changchun (Northeast China). Within each selected city, one comprehensive university was chosen. Subsequently, two colleges were conveniently sampled from each university, and two distinct majors were conveniently sampled from each college (aiming to cover the disciplines of humanities, sciences, engineering, and medicine). Finally, all students from three classes within each major were recruited to participate in the survey.

Inclusion criteria for study subjects were as follows: healthy college students aged 18–23, currently enrolled, with no history of smoking or alcohol abuse, and no known oral, endocrine, cardiac, renal, or other major chronic diseases (e.g., diabetes, hypertension, kidney disease, liver disease).

This study acknowledges the limitations of convenience sampling regarding representativeness and generalizability. Selecting only one university per geographic region means the sample may not fully represent all college students within that area, and conclusions should be extrapolated with caution.

### 2.3. Methods

#### 2.3.1. Sample Size Calculation

The data were categorical. During the analysis phase, post hoc pairwise comparisons (21 times in total) were conducted for the cross-sectional survey of water-related knowledge, attitudes, and practices (KAP) across the seven geographical divisions. The adjusted significance level was α = 0.05/21 = 0.0024. The following formula for sample size calculation was used:

$$n=\frac{2Z^{2}p(1-p)}{d^{2}}=\frac{2\times 2.81^{2}\times 0.151\times 0.849}{0.05^{2}}\approx 406$$

*p*: Based on the findings from a study of university students’ KAP regarding water intake at a university in Hebei Province [23], where the awareness rate of the recommended daily water intake (1500–1700 mL) was 15.1%, this study used this data as the reference for sample size calculation [26].

Given that this study employed multistage convenience sampling, a 10% increase was applied to the sample size for each stratum. The purpose was to enhance representativeness and account for potential attrition.

n=406÷0.9≈451

N=7n≈3157

Thus, approximately 451 participants were recruited from each region, and the total target sample size for the seven regions was 3157 participants. The study surveyed a total of 3215 participants.

#### 2.3.2. Questionnaire Survey

This study employed a Self-Designed Questionnaire on KAP Regarding Water Intake. The questionnaire was developed through systematic review and analysis of existing domestic and international questionnaires, followed by expert consultation and other steps [27]. It has been used in multiple field surveys [23,26,28]. The questionnaire comprises 26 items divided into four sections: Demographic Information (7 items, e.g., gender, age, ethnicity), Knowledge (6 items, e.g., daily recommended water intake, association between inadequate hydration and disease, perceived optimal timing for drinking water), Attitudes (8 items, e.g., willingness to change drinking habits, recognition of water’s importance for health), and Behavior (5 items, e.g., timing of water intake, methods of hydration). Responses are collected using either categorical or frequency options based on content. Prior to the survey launch, a standardized Investigator Manual was developed, and offline centralized training was conducted for site coordinators and investigators. Each participating institution conducted additional investigator training before the study commenced. All investigators underwent post-training assessments, with only qualified individuals participating in questionnaire administration. During the survey, professionally trained investigators guided and supervised respondents in completing questionnaires. Post-survey, investigators and quality control officers verified questionnaire completeness.

### 2.4. Data Processing and Statistical Analysis

Data were input into a database via Epi-Data 3.1 using double-data entry to ensure accuracy. Statistical analyses were conducted with SPSS 21.0. Categorical data were presented as percentages, calculated using these formulas:

Knowledge Awareness Rate = Number of respondents with correct answers to a question/Total number of respondents for that question × 100%(1)

Attitude Endorsement Rate = Number of respondents endorsing a specific attitude/Total number of respondents for that question × 100%(2)

Behavior Adoption Rate = Number of respondents practicing a specific behavior/Total number of respondents for that question × 100%(3)

Differences in responses across regions were assessed using chi-square tests. The overall significance level was set at *p* < 0.05 (two-tailed). To control for potential Type I error inflation when conducting multiple intergroup comparisons, the following strategy was adopted: First, overall chi-square tests were performed for all categorical variables across the seven major geographic regions, with a significance level set at α = 0.05. If the overall test yielded statistical significance *p* < 0.05, pairwise comparisons between subregions were then conducted. For these pairwise comparisons, the Bonferroni method was applied to adjust the significance level. Specifically, for a given indicator involving k comparisons, the adjusted significance level was set to α’ = 0.05/k. For example, when performing all possible pairwise comparisons among 7 partitions, k = 21. Unless otherwise specified, all instances of “*p* < 0.001” or specific *p*-values reported below refer to results adjusted by this method. To avoid potential misinterpretation from relying solely on *p*-values and to assess the practical significance of intergroup differences, all chi-square test results are supplemented with effect size reports. For 2 × 2 contingency tables, the Phi (φ) coefficient is reported; for higher-order contingency tables, Cramér’s V coefficient is reported.

### 2.5. Criteria for Excluding Invalid Questionnaires

A total of 3215 questionnaires were distributed, with 3215 returned. Exclusion criteria were as follows: (1) consistent pattern of responses throughout the questionnaire (e.g., identical selections for all items); (2) missing key information (e.g., age, gender); (3) missing responses in knowledge or attitude sections. Ultimately, 54 invalid questionnaires were excluded, yielding 3161 valid responses. The valid response rate was 98.3%.

## 3. Results

### 3.1. Demographic Characteristics of Participants

A total of 3215 questionnaires were collected. After removing incomplete/invalid ones, 3161 valid questionnaires remained, resulting in a 98.32% valid response rate. The geographical distribution was as follows: East China (Shanghai)—398 participants (12.6%), South China (Haikou)—517 (16.4%), North China (Tianjin)—317 (10.0%), Central China (Changsha)—500 (15.8%), Southwest China (Kunming)—458 (14.5%), Northwest China (Lanzhou)—485 (15.3%), and Northeast China (Changchun)—486 (15.4%). The sample included 1417 males and 1744 females, with a mean age of 19.8 years. Table 1 details the gender ratio by region and the proportion of each region in the sample.

### 3.2. Comparison of Awareness Rate of Water-Related Knowledge

From the overall perspective of China’s seven geographical divisions, college students demonstrated relatively high awareness rates regarding the correct way of water intake (water intake regularly and quantitatively, increasing the frequency of water intake/intake small amounts of water multiple times) at 78.8% (95% CI: 77.4%, 80.2%) and the appropriate types of water (plain boiled water or bottled water) at 82.8% (95% CI: 81.5%, 84.1%). However, the awareness rate of the key information that the recommended daily water intake is 1500–1700 mL was comparatively low at 21.1% (95% CI: 19.7%, 22.6%).

When it comes to the health issues potentially caused by insufficient water intake, college students showed relatively high awareness of “kidney stones” (61.5%, 95% CI: 59.8%, 63.2%), “constipation” (76.5%, 95% CI: 75.0%, 78.0%), and “dry skin” (80.6%, 95% CI: 79.2%, 82.0%). However, their awareness of the links between inadequate water intake and “hypertension” (25.7%, 95% CI: 24.2%, 27.3%), “coronary heart disease” (14.0%, 95% CI: 12.8%, 15.3%), “headache” (22.9%, 95% CI: 21.5%, 24.4%), “stroke” (10.2%, 9.2%, 11.3%), and “back pain” (12.1%, 95% CI: 11.0%, 13.3%) was comparatively low.

College students showed a high awareness (74.3%, 95% CI: 72.8%, 75.8%) of the health benefits of water intake on an empty stomach in the morning. However, fewer were aware of the benefits of intake after naps (33.9%, 95% CI: 32.3%, 35.6%), before bedtime (25.4%, 95% CI: 23.9%, 26.9%), after strenuous exercise (35.2%, 95% CI: 33.5%, 36.9%), and before meals (33.3%, 95% CI: 31.7%, 35.0%). Additionally, 37.9% (95% CI: 36.2%, 39.6%) of the students mistakenly believed that water intake only when thirsty is beneficial to health.

When comparing water intake awareness between genders via chi-square tests, it was found that females showed higher awareness than males in aspects like “water-intake method” (χ^2^ = 14.280, *p* < 0.001, φ = 0.067), “morning empty-stomach” (χ^2^ = 73.875, *p* < 0.001, φ = 0.153), “after bathing” (χ^2^ = 15.655, *p* < 0.001, φ = 0.070), and “before meals” (χ^2^ = 26.991, *p* < 0.001, φ = 0.092). On the other hand, males surpassed females in awareness of water intake “before bedtime” (χ^2^ = 45.960, *p* < 0.001, φ = 0.121), “after strenuous exercise” (χ^2^ = 3.972, *p* = 0.046, φ = 0.035), “during meals” (χ^2^ = 7.179, *p* = 0.008, φ = 0.048), “after meals” (χ^2^ = 12.958, *p* < 0.001, φ = 0.064), and “when feeling thirsty” (χ^2^ = 10.289, *p* = 0.002, φ = 0.057).

Chi-square tests revealed significant regional differences in college students’ awareness of correct water intake practices, the recommended daily water intake, appropriate water types, proper water intake times, and the links between insufficient water intake and diseases/symptoms (all *p* < 0.001). The awareness rates and 95% confidence interval of water-related knowledge of the surveyed subjects are shown in Table 2.

After corrected pairwise comparisons, it was found that significant regional differences emerged in water intake knowledge. South China (52.0%, 95% CI: 47.7%, 56.3%) had the lowest awareness of correct water intake practices (*p* < 0.001). For daily water intake recommendations, South China had the highest awareness (32.9%, 95% CI: 29.0%, 37.1%), while Southwest (14.8%, 95% CI: 11.8%, 18.4%) and Northeast China had lower awareness (10.9%, 95% CI: 8.4%, 13.9%) (*p* < 0.001).

In terms of awareness of proper water intake times for health benefits, college students in South China (54.7%, 95% CI: 50.4%, 59.0%) and Southwest China (66.6%, 95% CI: 62.2%, 70.8%) showed relatively low awareness of the benefits of morning empty-stomach water intake (*p* < 0.001). Students in Northwest China had a lower awareness of post-nap water intake (29.7%, 95% CI: 25.7%, 33.9%) compared to South China (39.3%, 95% CI: 35.1%, 43.6%) (*p* = 0.001). No significant differences were found in other regions. For pre-sleep intake, South China (39.3%, 95% CI: 35.1%, 43.6%) and Northeast China (31.5%, 95% CI: 27.5%, 35.7%) had higher awareness than other regions (*p* < 0.001). Regarding post-exercise intake, East China (42.5%, 95% CI: 37.7%, 47.4%), South China (41.8%, 95% CI: 37.6%, 46.1%), and Central China (37.8%, 95% CI: 33.6%, 42.2%) had higher awareness, while Southwest China (29.9%, 95% CI: 25.8%, 34.3%) and Northeast China (28.8%, 95% CI: 24.9%, 33.0%) had lower awareness (*p* < 0.001).

In terms of misconceptions about water intake timing, college students in South China had significantly higher rates of agreeing with the statements “water intake when feeling thirsty” (47.2%, 95% CI: 42.9%, 51.5%; *p* < 0.001) and “water intake whenever they remembered” (40.6%, 95% CI: 37.0–44.3%; *p* < 0.001) than those in other regions. Regarding the diseases or symptoms caused by insufficient water intake, South China students showed significantly higher awareness of “coronary heart disease” (25.9%, 95% CI: 22.3%, 29.8%) and “back pain” (22.2%, 95% CI: 18.8%, 25.9%) than other regions (*p* < 0.001). However, their awareness of the links between insufficient water intake and “kidney stones” (55.1%, 95% CI: 50.8%, 59.4%) and “constipation” (58.6%, 95% CI: 54.3%, 62.8%) was lower than in other regions (*p* < 0.001). Similarly, students in Southwest China (kidney stones: 53.3%, 95% CI: 48.7%, 57.9%; constipation: 73.6%, 95% CI: 69.4%, 77.5%) and Northeast China (kidney stones: 46.1%, 95% CI: 41.7%, 50.6%; constipation: 65.2%, 95% CI: 60.9%, 69.4%) also had lower awareness of these links. For the association between insufficient water intake and “dry skin,” students in South China (57.1%, 95% CI: 52.8%, 61.3%) and Northeast China (69.1%, 95% CI: 64.9%, 73.1%) had significantly lower awareness than other regions (*p* < 0.001). The pairwise comparison results of the surveyed subjects’ water-related knowledge awareness rates across the seven geographical divisions are shown in Table 3.

### 3.3. Comparison of Endorsement Rate of Water Intake-Related Attitudes

Overall, 92.6% (95% CI: 91.6%, 93.5%) of college students across the seven geographical divisions believed water intake is important for health. A total of 94.2% (95% CI: 93.3%, 95.0%) were willing to adjust their habits after understanding the health benefits of adequate hydration. A total of 87.9% (95% CI: 86.7%, 89.0%) showed interest in water-related knowledge and were open to water intake education. A total of 90.6% (95% CI: 89.5%, 91.6%) thought schools should conduct water intake knowledge activities. For preferred learning channels, 66.8% (95% CI: 65.1%, 68.4%) favored online platforms, and 45.6% (95% CI: 43.9%, 47.3%) opted for campus promotions or classroom education, while family only 19.1%.

Chi-square tests revealed significant differences among college students from different regions in terms of the aforementioned attitude items and preferences for knowledge acquisition channels (all *p* < 0.001). Table 4 presents details of participants’ attitudes toward water intake.

Corrected pairwise comparisons revealed that in South and Northeast China, college students had significantly lower endorsement rates for “water intake being important for health” (65.6% [61.5%, 69.6%] and 94.0% [91.8%, 95.7%], respectively) and “willingness to modify intake habits” (83.6% [80.2%, 86.6%] and 91.6% [89.0%, 93.7%]) compared to other regions (*p* < 0.001). In terms of preferred knowledge acquisition channels, South China students showed significant differences in using “lectures” (44.5%), “broadcasting” (45.5%), “family” (37.5%), and “peers” (38.5%) compared to other regions (*p* < 0.001). Additionally, students in South and Northeast China reported higher reliance on print materials (textbooks, newspapers, magazines) for water-related knowledge (*p* < 0.001) and lower usage of online platforms (58.0% [53.7%, 62.2%] and 56.4% [52.0%, 60.7%], respectively) than other regions (*p* < 0.001). Table 5 presents the pairwise comparison results of attitudes toward water intake across the seven geographical divisions.

### 3.4. Comparison of Adoption Rate of Water Intake-Related Behaviors

Overall, 56.0% (95% CI: 54.3%, 57.7%) of college students reported water intake on an empty stomach in the morning and 69.1% (95% CI: 67.5%, 70.7%) after strenuous exercise. However, 77.6% (95% CI: 76.1%, 79.0%) intake water when thirsty, 58.2% (95% CI: 56.5%, 59.9%) when they remember to, 44.8% during meals, and 47.4% after meals. In terms of water intake patterns, 50.1% (95% CI: 48.4%, 51.8%) intake water only when thirsty, while only 27.5% (95% CI: 26.0%, 29.1%) follow a regular intake pattern.

Chi-square tests revealed no significant regional differences in water intake after naps but significant differences (*p* < 0.05) in other intake times and correct intake patterns. Details are in Table 6.

Pairwise comparisons showed that Northeast and South China had lower rates of water intake when thirsty (70.8% [66.7%, 74.7%] and 61.3% [57.0%, 65.5%]) than other regions except the southwest region (*p* < 0.001). South China had lower rates of morning empty-stomach water intake (49.5% [45.2%, 53.8%]) than Northwest and Northeast China (59.6% [55.1%, 64.0%] and 59.5% [55.1%, 63.7%], *p* = 0.001). For pre-sleep water intake, South and Northeast China had higher rates (47.4% [43.1%, 51.7%] and 45.5% [41.1%, 49.9%]) than Central and Southwest China (34.6% [30.5%, 38.9%] and 35.4% [31.1%, 39.9%], *p* < 0.001), while North China had lower rates (36.3%, [31.1%, 41.8]) than South China. South China’s post-exercise water intake rate (57.1%, [52.8%, 61.3%]) was lower than most regions (*p* < 0.001). Southwest China had the lowest post-bath water intake rate (21.6% [18.0%, 25.6%], *p* < 0.001). South and Northeast China had higher pre-meal water intake rates (40.6% [36.5%, 44.9%] and 35.2% [31.1%, 39.5%]) than other regions except the northwest region (*p* < 0.001). Central China had lower rates of regular and quantitative water intake (20.4%, [17.1%, 24.1%]) than South and Northwest China (31.1% [27.2%, 35.2%] and 49.4% [44.9%, 53.9%]) (*p* < 0.001), and Northeast China (23.7% [20.2%, 27.6%]) had lower rates than Northwest China (33.8% [29.7–38.1%], *p* = 0.001). Table 7 presents the pairwise comparison results.

## 4. Discussion

This study represents the first systematic investigation of Chinese university students’ knowledge, attitudes, and practices regarding drinking water within the framework of seven major geographical regions nationwide. Core findings reveal significant geographical variations in students’ drinking water behaviors, particularly in knowledge accuracy and behavioral patterns, which exhibit distinct regional characteristics. These differences not only reflect the influence of local environments and cultures but also suggest that health behavior interventions require regional specificity. The following interpretation of key findings is grounded in the KAP theoretical framework.

### 4.1. Key Findings and Interpretation of Geographic Variations

This study reveals that the core knowledge gap regarding water consumption lies in the generally low awareness of recommended daily intake (21.1%) and insufficient recognition of the link between inadequate hydration and certain chronic diseases (e.g., hypertension, coronary heart disease). This provides a weak knowledge foundation for attitude transformation and behavioral practice. Although attitudes are generally positive, students in South China and Northeast China exhibit significantly lower recognition of water’s importance and willingness to change behaviors. This may stem from entrenched local dietary and drinking habits reducing perceived need for change. Behaviorally, reliance on thirst signals, inconsistent drinking habits, and low adoption of proper patterns (27.5% for scheduled and measured intake) are widespread issues, indicating knowledge fails to translate effectively into practice.

Significant geographic disparities exist in university students’ knowledge, belief, and behavior regarding water consumption. These differences are not coincidental but result from the complex interplay of multiple factors, including climate, regional culture, dietary traditions, and the prevalence of health education. Students in South China exhibit unique patterns across multiple knowledge items, such as the lowest awareness of proper hydration methods (52.0%) coupled with the highest prevalence of the misconception that “drinking when thirsty” is sufficient (47.2%). Conversely, students in the northwest region demonstrated superior knowledge retention and consistent hydration practices. These disparities likely stem from the interplay of climate, regional culture, and dietary traditions. For instance, the deeply ingrained “Lingnan dietary pattern” emphasizes “frequent tea consumption and regular soup preparation.” Tea and soup occupy a significant role in daily fluid intake, potentially substituting for the need to drink plain water regularly. This may contribute to relatively low awareness of standardized knowledge like “drinking water at fixed times and quantities,” while also shaping unique hydration rhythms (e.g., replenishing fluids with soup during or after meals) [29,30]. Geographical disparities manifest not only in natural and cultural dimensions but also in the distribution of social resources. The 2024 National Health Literacy Monitoring Data reveals a gradient difference in health literacy levels among residents in eastern, central, and western regions (34.98%, 31.48%, and 27.27%, respectively). This regional imbalance in overall health information access and literacy likely permeates specific health topics like “water consumption,” affecting the breadth and depth of knowledge dissemination. This helps explain why students in the equally arid northwest (high awareness rate) and northeast (low awareness rate) regions exhibit differing levels of understanding regarding the link between water intake and health.

### 4.2. Barrier Analysis Based on KAP Theory

According to KAP theory, the formation of health behaviors follows a “knowledge → attitude → behavior” pathway [19,20]. This study identified a significant disconnect within this pathway. Although most students held positive attitudes toward drinking water, their weak knowledge base—particularly regarding quantified recommended values—may have hindered the development of firm, concrete change beliefs. More critically, translating positive attitudes into consistent behaviors faces contextual and habitual barriers. For instance, the deeply ingrained habit of “drinking only when thirsty” contradicts scientific recommendations for proactive, timed hydration. Altering this requires strong self-regulation and environmental influence. Furthermore, regional social norms and physical environments (such as the accessibility of drinking water on campus) may subtly shape and reinforce specific drinking patterns, making it difficult for individual knowledge to counteract powerful environmental and habitual forces.

### 4.3. Strengths, Limitations, and Future Directions

The primary strength of this study lies in its pioneering nationwide survey of college students’ knowledge, beliefs, and practices regarding water consumption across multiple geographic regions, providing baseline data for understanding regional variations in this health behavior. However, several limitations exist: First, the use of multistage convenience sampling, coupled with selecting only one representative university per region, limits sample representativeness, necessitating caution when extrapolating results. Second, the cross-sectional design precludes establishing causal relationships among knowledge, belief, and behavior. Third, the assessment of drinking behavior relies on self-reporting, which may introduce recall bias and social desirability bias.

Future studies may employ stratified random sampling to enhance sample representativeness and incorporate more granular environmental variables (e.g., campus drinking water facilities, class schedules) and socioeconomic factors for multilevel analysis. Longitudinal or interventional studies could help elucidate causal pathways between knowledge, belief, and behavior. Furthermore, developing and validating standardized drinking water assessment tools applicable to national cultural contexts would significantly improve research comparability.

## 5. Conclusions

In conclusion, the knowledge, attitude, and practice (KAP) status regarding drinking water among Chinese university students is not optimistic and exhibits significant geographical variation. Key issues include knowledge gaps, disconnects between attitudes and behaviors, and deeply ingrained unscientific drinking habits. Analysis grounded in KAP theory indicates that future health promotion strategies must transcend mere knowledge dissemination, adopting region-specific, multi-tiered comprehensive interventions. Building upon general education, tailored information should address regional knowledge gaps and cultural characteristics—such as correcting cognitive biases regarding water intake levels in South China and strengthening public science communication on the health–water connection in Northeast China. Simultaneously, campus environmental support (e.g., upgrading drinking facilities, installing hydration reminders) and habit-forming strategies are crucial for converting attitudes into behaviors. These measures aim to help college students establish proactive, adequate, and regular scientific drinking habits, thereby enhancing their long-term health outcomes.

## Figures and Tables

**Table 1:** Demographic characteristics of participants by region and the proportion of each region.

Region	Gender (% Within Region)		N * (%)
	Male	Female	
East China	132 (33.2)	266 (66.8)	398 (12.6)
South China	251 (48.5)	266 (51.5)	517 (16.4)
North China	117 (36.9)	200 (63.1)	317 (10.0)
Central China	250 (50.0)	250 (50.0)	500 (15.8)
Southwest China	186 (40.6)	272 (59.4)	458 (14.5)
Northwest China	240 (49.5)	245 (50.5)	485 (15.3)
Northeast China	241 (49.6)	245 (50.4)	486 (15.4)
Total	1417 (44.8)	1744 (55.2)	3161 (100)

* In the parentheses is the proportion of this region among the total population.

**Table 2:** The awareness rate of water intake knowledge among the survey subjects.

		East China (n = 398) Percentage (95%CI)	South China (n = 517) Percentage (95%CI)	North China (n = 317) Percentage (95%CI)	Central China (n = 500) Percentage (95%CI)	Southwest China (n = 458) Percentage (95%CI)	Northwest China (n = 485) Percentage (95%CI)	Northeast China (n = 486) Percentage (95%CI)	Overall *p* (Formatted)	Effect Size	Male (n = 1417) Percentage (95%CI)	Female (n = 1744) Percentage (95%CI)	Overall *p* (Formatted)	Effect Size
**Correct water intake method**		91.5% (88.3–93.8)	52.0% (47.7–56.3)	91.8% (88.3–94.3)	89.0% (86.0–91.5)	77.3% (73.2–80.9)	88.2% (85.1–90.8)	69.8% (65.5–73.7)	<0.001	0.344	75.7% (73.4%, 77.9%)	81.3% (79.4%, 83.1%)	<0.001	0.067
**Correct daily water intake**		24.6% (20.6–29.1)	32.9% (29.0–37.0)	16.7% (13.0–21.2)	24.4% (20.8–28.4)	14.8% (11.9–18.4)	21.2% (17.8–25.1)	10.9% (8.4–14.0)	<0.001	0.120	20.8% (18.8%, 22.9%)	21.3% (19.4%, 23.3%)	0.726	0.006
**Suitable water intake time**	**Morning empty stomach**	86.2% (82.4–89.2)	54.7% (50.4–59.0)	78.9% (74.0–83.0)	81.0% (77.3–84.2)	66.6% (62.2–70.8)	82.7% (79.1–85.8)	74.5% (70.4–78.2)	<0.001	0.239	66.9% (64.4%, 69.3%)	80.3% (78.3%, 82.1%)	<0.001	0.153
	**After naps**	30.2% (25.8–34.8)	39.3% (35.1–43.5)	36.6% (31.5–42.0)	32.8% (28.8–37.0)	33.4% (29.2–37.8)	29.7% (25.8–33.9)	35.2% (31.1–39.5)	0.023	0.068	33.5% (31.1%, 36.0%)	34.2% (32.0%, 36.5%)	0.645	0.008
	**Before sleep**	19.3% (15.8–23.5)	39.3% (35.1–43.5)	17.7% (13.9–22.2)	22.2% (18.8–26.0)	21.6% (18.1–25.6)	21.2% (17.8–25.1)	31.5% (27.5–35.7)	<0.001	0.169	31.2% (28.9%, 33.7%)	20.6% (18.7%, 22.6%)	<0.001	0.121
	**After strenuous exercise**	42.5% (37.7–47.4)	41.8% (37.6–46.1)	34.1% (29.1–39.4)	37.8% (33.7–42.1)	29.9% (25.9–34.3)	32.0% (28.0–36.2)	28.8% (25.0–33.0)	<0.001	0.108	37.1% (34.6%, 39.7%)	33.7% (31.5%, 36.0%)	0.046	0.035
	**After bathing**	34.4% (29.9–39.2)	39.7% (35.5–43.9)	35.6% (30.6–41.1)	30.8% (26.9–35.0)	25.1% (21.4–29.3)	30.9% (27.0–35.2)	27.0% (23.2–31.1)	<0.001	0.103	28.2% (25.9%, 30.6%)	34.7% (32.5%, 37.0%)	<0.001	0.07
	**Before meals**	35.4% (30.9–40.2)	38.5% (34.4–42.8)	31.2% (26.4–36.5)	28.0% (24.2–32.1)	31.0% (26.9–35.4)	36.9% (32.7–41.3)	31.7% (27.7–36.0)	0.005	0.077	28.5% (26.2%, 30.9%)	37.3% (35.0%, 39.6%)	<0.001	0.092
	**During meals**	9.8% (7.3–13.1)	33.5% (29.5–37.6)	13.9% (10.5–18.1)	14.2% (11.4–17.5)	16.4% (13.3–20.0)	21.9% (18.4–25.7)	27.2% (23.4–31.3)	<0.001	0.196	22.4% (20.3%, 24.7%)	18.5% (16.7%, 20.4%)	0.007	0.048
	**After meals**	22.1% (18.3–26.4)	44.9% (40.6–49.2)	28.1% (23.4–33.3)	22.4% (19.0–26.3)	30.1% (26.1–34.5)	25.6% (21.9–29.6)	31.7% (27.7–36.0)	<0.001	0.165	32.9% (30.5%, 35.4%)	27.0% (25.0%, 29.1%)	<0.001	0.064
	**Feeling thirsty**	32.7% (28.2–37.4)	47.2% (42.9–51.5)	34.1% (29.1–39.4)	39.0% (34.8–43.3)	36.9% (32.6–41.4)	35.5% (31.3–39.8)	37.2% (33.1–41.6)	<0.001	0.093	41.0% (38.4%, 43.6%)	35.4% (33.2%, 37.7%)	0.001	0.057
	**When remember**	29.6% (25.4–34.3)	40.6% (36.5–44.9)	29.7% (24.9–34.9)	25.0% (21.4–29.0)	27.5% (23.6–31.8)	27.8% (24.0–32.0)	24.5% (20.9–28.5)	<0.001	0.117	28.2% (25.9%, 30.6%)	30.3% (28.1%, 32.5%)	0.194	0.023
**Suitable water types**		86.9% (83.3–89.9)	52.6% (48.3–56.9)	89.3% (85.4–92.2)	92.0% (89.3–94.1)	88.2% (84.9–90.9)	90.5% (87.6–92.8)	84.8% (81.3–87.7)	<0.001	0.358	82.1% (80.0%, 84.0%)	83.3% (81.5%, 85.0%)	0.359	0.016
**Diseases or symptoms related to insufficient water intake**	**Stroke**	11.3% (8.6–14.8)	15.9% (13.0–19.3)	3.8% (2.2–6.5)	11.6% (9.1–14.7)	8.3% (6.1–11.2)	5.2% (3.5–7.5)	13.0% (10.3–16.2)	<0.001	0.130	10.9% (9.4%, 12.7%)	9.7% (8.4%, 11.2%)	0.277	0.019
	**Hypertension**	29.6% (25.4–34.3)	29.4% (25.6–33.5)	26.8% (22.2–31.9)	31.4% (27.5–35.6)	20.1% (16.7–24.0)	22.9% (19.4–26.8)	20.2% (16.8–24.0)	<0.001	0.102	27.9% (25.6%, 30.3%)	23.9% (22.0%, 26.0%)	0.01	0.046
	**Coronary disease**	10.3% (7.7–13.7)	25.9% (22.3–29.9)	9.5% (6.7–13.2)	15.6% (12.7–19.0)	8.5% (6.3–11.4)	13.0% (10.3–16.3)	12.1% (9.5–15.3)	<0.001	0.164	14.6% (12.8%, 16.6%)	13.6% (12.0%, 15.3%)	0.412	0.015
	**Kidney stone**	75.9% (71.4–79.8)	55.1% (50.8–59.4)	67.2% (61.8–72.1)	71.2% (67.1–75.0)	53.3% (48.7–57.8)	66.0% (61.7–70.1)	46.1% (41.7–50.5)	<0.001	0.206	58.9% (56.3%, 61.4%)	63.6% (61.3%, 65.8%)	0.006	0.049
	**Constipation**	86.4% (82.7–89.5)	58.6% (54.3–62.8)	88.6% (84.7–91.7)	88.6% (85.5–91.1)	73.6% (69.4–77.4)	80.8% (77.1–84.1)	65.2% (60.9–69.3)	<0.001	0.265	72.3% (69.9%, 74.6%)	79.8% (77.9%, 81.6%)	<0.001	0.088
	**Dry skin**	89.4% (86.0–92.1)	57.1% (52.8–61.3)	90.2% (86.5–93.0)	92.0% (89.3–94.1)	85.8% (82.3–88.7)	87.0% (83.7–89.7)	69.1% (64.9–73.1)	<0.001	0.321	75.9% (73.6%, 78.1%)	84.5% (82.8%, 86.1%)	<0.001	0.108
	**Headache**	21.1% (17.4–25.4)	26.9% (23.2–30.9)	15.5% (11.9–19.8)	24.8% (21.2–28.8)	24.0% (20.3–28.1)	21.9% (18.4–25.7)	23.3% (19.7–27.2)	0.009	0.073	24.5% (22.3%, 26.8%)	21.7% (19.8%, 23.7%)	0.061	0.033
	**Back pain**	8.0% (5.8–11.1)	22.2% (18.9–26.0)	5.4% (3.4–8.4)	11.2% (8.7–14.3)	11.4% (8.8–14.6)	10.9% (8.5–14.0)	11.7% (9.2–14.9)	<0.001	0.150	12.5% (10.8%, 14.4%)	11.8% (10.3%, 13.4%)	0.527	0.011
	**Others**	1.8% (0.9–3.6)	0.2% (0.0–1.1)	0.9% (0.3–2.7)	2.2% (1.2–3.9)	2.8% (1.7–4.8)	1.4% (0.7–2.9)	2.9% (1.7–4.8)	0.014	0.071	2.0% (1.4%, 2.9%)	1.6% (1.1%, 2.3%)	0.432	0.014

*p* < 0.05 indicates that the awareness rates of water intake knowledge among different groups are not completely the same, and there are statistically significant differences.

**Table 3:** Pairwise comparison of the awareness rate of water intake knowledge among the survey subjects (*p*-value and Cramér’s V).

AREA		E						S					N				C			SW		NW
AREA *		S	N	C	SW	NW	NE	N	C	SW	NW	NE	C	SW	NW	NE	SW	NW	NE	NW	NE	NE
**Correct water intake method**		<0.001, 0.423	0.870, 0.006	0.221, 0.041	<0.001, 0.192	0.119, 0.053	<0.001, 0.268	<0.001, 0.411	<0.001, 0.404	<0.001, 0.262	<0.001, 0.393	<0.001, 0.181	0.192, 0.046	<0.001, 0.191	0.107, 0.057	<0.001, 0.262	<0.001, 0.157	0.710, 0.012	<0.001, 0.238	<0.001, 0.146	0.009, 0.085	<0.001, 0.227
**Correct daily water intake**		0.006, 0.090	0.010, 0.096	0.938, 0.003	<0.001, 0.123	0.232, 0.040	<0.001, 0.181	<0.001, 0.177	0.003, 0.094	<0.001, 0.210	<0.001, 0.131	<0.001, 0.264	0.009, 0.091	0.480, 0.025	0.114, 0.056	0.017, 0.084	<0.001, 0.120	0.237, 0.038	<0.001, 0.177	0.011, 0.083	0.070, 0.059	<0.001, 0.141
**Suitable water intake time**	**Morning empty stomach**	<0.001, 0.335	0.010, 0.097	0.039, 0.069	<0.001, 0.228	0.155, 0.048	<0.001, 0.145	<0.001, 0.244	<0.001, 0.281	<0.001, 0.121	<0.001, 0.300	<0.001, 0.206	0.456, 0.026	<0.001, 0.134	0.177, 0.048	0.154, 0.050	<0.001, 0.164	0.494, 0.022	0.014, 0.078	<0.001, 0.185	0.008, 0.087	0.002, 0.100
	**After naps**	0.004, 0.095	0.069, 0.068	0.396, 0.028	0.308, 0.035	0.882, 0.005	0.113, 0.053	0.441, 0.027	0.032, 0.067	0.058, 0.061	0.001, 0.101	0.182, 0.042	0.266, 0.039	0.360, 0.033	0.041, 0.072	0.684, 0.014	0.842, 0.006	0.293, 0.034	0.429, 0.025	0.220, 0.040	0.565, 0.019	0.067, 0.059
	**Before sleep**	<0.001, 0.214	0.566, 0.021	0.296, 0.035	0.413, 0.028	0.488, 0.023	<0.001, 0.138	<0.001, 0.227	<0.001, 0.185	<0.001, 0.190	<0.001, 0.196	0.010, 0.081	0.117, 0.055	0.176, 0.049	0.215, 0.044	<0.001, 0.154	0.827, 0.007	0.714, 0.012	0.001, 0.105	0.887, 0.005	0.001, 0.111	<0.001, 0.116
	**After strenuous exercise**	0.836, 0.007	0.022, 0.086	0.156, 0.047	<0.001, 0.131	0.001, 0.108	<0.001, 0.142	0.027, 0.077	0.195, 0.041	<0.001, 0.123	0.001, 0.102	<0.001, 0.135	0.280, 0.038	0.221, 0.044	0.534, 0.022	0.115, 0.056	0.010, 0.083	0.055, 0.061	0.003, 0.095	0.497, 0.022	0.709, 0.012	0.286, 0.034
	**After bathing**	0.105, 0.054	0.733, 0.013	0.249, 0.038	0.003, 0.102	0.270, 0.037	0.016, 0.081	0.248, 0.040	0.003, 0.093	<0.001, 0.155	0.004, 0.091	<0.001, 0.134	0.150, 0.050	0.002, 0.114	0.164, 0.049	0.009, 0.092	0.050, 0.063	0.965, 0.001	0.183, 0.042	0.047, 0.065	0.519, 0.021	0.172, 0.044
	**Before meals**	0.342, 0.031	0.238, 0.044	0.017, 0.080	0.170, 0.047	0.649, 0.015	0.241, 0.039	0.034, 0.074	<0.001, 0.111	0.014, 0.078	0.605, 0.016	0.024, 0.071	0.323, 0.035	0.947, 0.002	0.099, 0.058	0.892, 0.005	0.308, 0.033	0.003, 0.095	0.206, 0.040	0.056, 0.062	0.821, 0.007	0.087, 0.055
	**During meals**	<0.001, 0.278	0.091, 0.063	0.046, 0.067	0.005, 0.097	<0.001, 0.162	<0.001, 0.219	<0.001, 0.217	<0.001, 0.226	<0.001, 0.196	<0.001, 0.129	0.030, 0.068	0.898, 0.004	0.343, 0.034	0.005, 0.100	<0.001, 0.157	0.349, 0.030	0.002, 0.100	<0.001, 0.160	0.033, 0.070	<0.001, 0.130	0.055, 0.062
	**After meals**	<0.001, 0.237	0.066, 0.069	0.918, 0.003	0.008, 0.091	0.232, 0.040	0.001, 0.107	<0.001, 0.168	<0.001, 0.237	<0.001, 0.152	<0.001, 0.202	<0.001, 0.135	0.066, 0.064	0.536, 0.022	0.432, 0.028	0.276, 0.038	0.006, 0.088	0.244, 0.037	0.001, 0.105	0.118, 0.051	0.605, 0.017	0.035, 0.068
	**Feeling thirsty**	<0.001, 0.147	0.692, 0.015	0.050, 0.066	0.195, 0.044	0.383, 0.029	0.156, 0.048	<0.001, 0.129	0.008, 0.083	0.001, 0.104	<0.001, 0.119	0.001, 0.101	0.155, 0.050	0.419, 0.029	0.685, 0.014	0.360, 0.032	0.503, 0.022	0.251, 0.037	0.570, 0.018	0.647, 0.015	0.913, 0.004	0.564, 0.018
	**When remember**	0.001, 0.113	1.000, 0.000	0.119, 0.052	0.490, 0.024	0.553, 0.020	0.085, 0.058	0.001, 0.111	<0.001, 0.166	<0.001, 0.138	<0.001, 0.134	<0.001, 0.172	0.143, 0.051	0.516, 0.023	0.577, 0.020	0.105, 0.057	0.377, 0.029	0.313, 0.032	0.852, 0.006	0.911, 0.004	0.289, 0.034	0.235, 0.038
**Suitable water types**		<0.001, 0.363	0.339, 0.036	0.013, 0.083	0.572, 0.019	0.092, 0.057	0.361, 0.031	<0.001, 0.377	<0.001, 0.438	<0.001, 0.385	<0.001, 0.417	<0.001, 0.345	0.186, 0.046	0.646, 0.017	0.566, 0.020	0.068, 0.064	0.049, 0.064	0.409, 0.026	<0.001, 0.113	0.250, 0.037	0.123, 0.050	0.007, 0.087
**Diseases or symptoms related to insufficient water intake**	**Stroke**	0.048, 0.065	<0.001, 0.138	0.891, 0.005	0.138, 0.051	0.001, 0.113	0.454, 0.025	<0.001, 0.185	0.049, 0.062	<0.001, 0.115	<0.001, 0.173	0.192, 0.041	<0.001, 0.136	0.012, 0.090	0.366, 0.032	<0.001, 0.154	0.089, 0.055	<0.001, 0.116	0.514, 0.021	0.053, 0.063	0.020, 0.075	<0.001, 0.136
	**Hypertension**	0.007, 0.003	0.404, 0.031	0.572, 0.019	0.001, 0.111	0.023, 0.077	0.001, 0.110	0.421, 0.028	0.488, 0.022	0.001, 0.107	0.019, 0.074	0.001, 0.107	0.162, 0.049	0.028, 0.079	0.206, 0.045	0.028, 0.077	<0.001, 0.129	0.003, 0.096	<0.001, 0.128	0.296, 0.034	0.976, 0.001	0.302, 0.033
	**Coronary disease**	<0.001, 0.197	0.710, 0.014	0.020, 0.078	0.371, 0.031	0.218, 0.041	0.391, 0.029	<0.001, 0.201	<0.001, 0.127	<0.001, 0.227	<0.001, 0.163	<0.001, 0.175	0.012, 0.088	0.649, 0.016	0.127, 0.054	0.238, 0.042	0.001, 0.108	0.242, 0.037	0.116, 0.050	0.027, 0.072	0.068, 0.059	0.690, 0.013
	**Kidney stone**	<0.001, 0.215	0.010, 0.096	0.115, 0.053	<0.001, 0.235	0.001, 0.108	<0.001, 0.302	0.001, 0.119	<0.001, 0.166	0.563, 0.019	<0.001, 0.111	0.004, 0.090	0.225, 0.042	<0.001, 0.139	0.722, 0.013	<0.001, 0.207	<0.001, 0.185	0.077, 0.056	<0.001, 0.255	<0.001, 0.130	0.027, 0.072	<0.001, 0.200
	**Constipation**	<0.001, 0.303	0.376, 0.033	0.327, 0.033	<0.001, 0.159	0.026, 0.075	<0.001, 0.243	<0.001, 0.318	<0.001, 0.339	<0.001, 0.157	<0.001, 0.241	0.031, 0.068	0.985, 0.001	<0.001, 0.184	0.003, 0.104	<0.001, 0.263	<0.001, 0.193	0.001, 0.108	<0.001, 0.278	0.008, 0.086	0.005, 0.091	<0.001, 0.176
	**Dry skin**	<0.001, 0.354	0.734, 0.013	0.187, 0.044	0.108, 0.055	0.266, 0.037	<0.001, 0.245	<0.001, 0.350	<0.001, 0.399	<0.001, 0.315	<0.001, 0.332	<0.001, 0.125	0.379, 0.031	0.067, 0.066	0.167, 0.049	<0.001, 0.247	0.002, 0.099	0.011, 0.082	<0.001, 0.290	0.590, 0.018	<0.001, 0.199	<0.001, 0.216
	**Headache**	0.043, 0.067	0.054, 0.072	0.192, 0.044	0.310, 0.035	0.787, 0.009	0.446, 0.026	<0.001, 0.133	0.448, 0.024	0.305, 0.033	0.064, 0.058	0.185, 0.042	0.001, 0.111	0.004, 0.104	0.025, 0.079	0.007, 0.095	0.778, 0.009	0.275, 0.035	0.569, 0.018	0.430, 0.026	0.782, 0.009	0.603, 0.017
	**Back pain**	<0.001, 0.192	0.159, 0.053	0.114, 0.053	0.104, 0.056	0.148, 0.049	0.070, 0.061	<0.001, 0.224	<0.001, 0.148	<0.001, 0.144	<0.001, 0.151	<0.001, 0.139	0.004, 0.100	0.004, 0.103	0.006, 0.096	0.002, 0.108	0.940, 0.002	0.892, 0.004	0.795, 0.008	0.835, 0.007	0.857, 0.006	0.694, 0.013
	**Others**	0.024, 0.083	0.358, 0.034	0.639, 0.016	0.297, 0.036	0.709, 0.013	0.276, 0.037	0.127, 0.053	0.003, 0.093	0.001, 0.111	0.026, 0.070	<0.001, 0.111	0.178, 0.047	0.069, 0.065	0.535, 0.022	0.063, 0.066	0.528, 0.020	0.375, 0.028	0.497, 0.022	0.137, 0.048	0.969, 0.001	0.124, 0.049

* E represents East China; S represents South China; N represents North China; C represents Central China; SW represents Southwest China; NW represents Northwest China; NE represents Northeast China. *p* < 0.002, which is the level of one-sided test, indicates that when making pairwise comparisons among different geographical intervals, there are statistically significant differences in the awareness rates of water intake knowledge.

**Table 4:** The endorsement rate of water intake attitudes among the survey subjects.

Demographic Indicators		Important	Willing to Change Water Intake Behavior	Interested	Willing to Follow	Support Promotional Campaigns	Textbooks	Campus Promotion	Lectures	TV/Radio	Magazine	Internet	Family	Peers	Others
Sex	Male	92.4% (91.0–93.7)	93.9% (92.6–95.0)	85.9% (84.0–87.6)	89.2% (87.5–90.7)	90.1% (88.4–91.6)	28.1% (25.8–30.5)	44.0% (41.4–46.6)	27.0% (24.7–29.4)	26.8% (24.5–29.2)	25.7% (23.4–28.1)	65.0% (62.5–67.5)	19.2% (17.2–21.3)	21.7% (19.6–24.0)	0.6% (0.3–1.1)
	Female	92.7% (91.4–93.8)	94.5% (93.4–95.5)	89.6% (88.1–91.0)	91.7% (90.4–93.0)	90.4% (89.0–91.8)	26.0% (23.9–28.2)	46.9% (44.6–49.3)	30.6% (28.4–32.8)	31.1% (29.0–33.4)	26.7% (24.7–28.8)	68.3% (66.1–70.5)	19.0% (17.2–21.0)	21.7% (19.8–23.7)	0.5% (0.3–1.0)
*p*		0.051	0.447	0.014	0.015	0.774	0.183	0.099	0.026	0.008	0.512	0.046	0.910	0.967	0.853
**Effect size**		0.004	0.014	0.063	0.043	0.005	0.024	0.029	0.040	0.047	0.012	0.035	0.002	0.001	0.003
Area *	**East China (n = 398) percentage (95%CI)**	98.5% (96.8–99.3)	96.5% (94.2–97.9)	91.0% (87.7–93.4)	90.2% (86.9–92.7)	88.9% (85.5–91.7)	20.1% (16.5–24.3)	44.5% (39.7–49.4)	29.6% (25.4–34.3)	26.4% (22.3–30.9)	17.3% (13.9–21.4)	74.6% (70.1–78.6)	11.3% (8.6–14.8)	12.6% (9.7–16.2)	0.5% (0.1–1.8)
	**South China (n = 517) percentage (95%CI)**	65.6% (61.4–69.5)	83.6% (80.1–86.5)	80.9% (77.2–84.0)	86.8% (83.7–89.5)	87.6% (84.5–90.2)	42.6% (38.4–46.9)	52.2% (47.9–56.5)	44.5% (40.3–48.8)	45.5% (41.2–49.8)	40.6% (36.5–44.9)	58.0% (53.7–62.2)	37.5% (33.5–41.8)	38.5% (34.4–42.8)	0.0% (0.0–0.7)
	**North China (n = 317) percentage (95%CI)**	99.7% (98.2–99.9)	97.5% (95.1–98.7)	94.6% (91.6–96.6)	92.7% (89.3–95.1)	93.4% (90.1–95.6)	19.9% (15.9–24.6)	41.3% (36.0–46.8)	27.1% (22.5–32.3)	21.8% (17.6–26.6)	21.1% (17.0–26.0)	68.5% (63.1–73.3)	13.6% (10.2–17.8)	14.2% (10.8–18.5)	0.0% (0.0–1.2)
	**Central China (n = 500) percentage (95%CI)**	98.8% (97.4–99.4)	98.0% (96.4–98.9)	90.0% (87.1–92.3)	87.2% (84.0–89.8)	90.0% (87.1–92.3)	22.6% (19.2–26.5)	42.6% (38.3–47.0)	21.8% (18.4–25.6)	23.8% (20.3–27.7)	18.2% (15.1–21.8)	74.8% (70.8–78.4)	14.6% (11.8–18.0)	20.2% (16.9–23.9)	0.6% (0.2–1.7)
	**Southwest China (n = 458) percentage (95%CI)**	97.6% (95.8–98.7)	95.9% (93.6–97.3)	85.8% (82.3–88.7)	93.7% (91.1–95.6)	91.9% (89.1–94.1)	20.7% (17.3–24.7)	42.1% (37.7–46.7)	29.5% (25.5–33.8)	26.6% (22.8–30.9)	23.6% (19.9–27.7)	69.9% (65.5–73.9)	13.8% (10.9–17.2)	15.7% (12.7–19.3)	1.5% (0.7–3.1)
	**Northwest China (n = 485) percentage (95%CI)**	99.2% (97.9–99.7)	98.8% (97.3–99.4)	93.2% (90.6–95.1)	95.3% (93.0–96.8)	95.3% (93.0–96.8)	23.7% (20.1–27.7)	48.0% (43.6–52.5)	24.5% (20.9–28.6)	27.8% (24.0–32.0)	26.6% (22.9–30.7)	68.2% (64.0–72.2)	19.0% (15.7–22.7)	21.4% (18.0–25.3)	0.2% (0.0–1.2)
	**Northeast China (n = 486) percentage (95%CI)**	94.0% (91.6–95.8)	91.6% (88.8–93.7)	83.3% (79.8–86.4)	89.5% (86.5–91.9)	86.0% (82.6–88.8)	34.0% (29.9–38.3)	46.1% (41.7–50.5)	24.3% (20.7–28.3)	28.4% (24.6–32.6)	32.1% (28.1–36.4)	56.4% (51.9–60.7)	19.3% (16.1–23.1)	23.7% (20.1–27.6)	0.8% (0.3–2.1)
*p*		<0.001	<0.001	<0.001	<0.001	<0.001	<0.001	0.010	<0.001	<0.001	<0.001	<0.001	<0.001	<0.001	14.693
**Effect size**		0.460	0.223	0.146	0.106	0.103	0.187	0.073	0.163	0.164	0.180	0.149	0.218	0.201	0.023

* *p* < 0.05 indicates that the attitude endorsement rates among different groups are not completely the same, and there are statistically significant differences.

**Table 5:** Pairwise comparison of the endorsement rates of water intake attitudes among the survey subjects in different regions (*p*-value and Cramér’s V).

AREA	E						S					N				C			SW		NW
AREA *	S	N	C	SW	NW	NE	N	C	SW	NW	NE	C	SW	NW	NE	SW	NW	NE	NW	NE	NE
Important	<0.001, 0.407	0.108, 0.060	0.690, 0.013	0.350, 0.032	0.340, 0.032	0.001, 0.114	<0.001, 0.403	<0.001, 0.432	<0.001, 0.404	<0.001, 0.436	<0.001, 0.351	0.181, 0.047	0.021, 0.083	0.370, 0.032	<0.001, 0.146	0.159, 0.045	0.557, 0.019	<0.001, 0.129	0.053, 0.063	0.007, 0.088	<0.001, 0.142
Willing to change	<0.001, 0.206	0.445, 0.029	0.161, 0.047	0.633, 0.016	0.023, 0.076	0.003, 0.101	<0.001, 0.215	<0.001, 0.248	<0.001, 0.199	<0.001, 0.264	<0.001, 0.121	0.619, 0.017	0.225, 0.044	0.174, 0.048	0.001, 0.121	0.053, 0.063	0.344, 0.030	<0.001, 0.145	0.005, 0.091	0.007, 0.088	<0.001, 0.168
Interested	<0.001, 0.141	0.062, 0.070	0.629, 0.016	0.020, 0.080	0.217, 0.042	0.001, 0.112	<0.001, 0.193	<0.001, 0.129	0.039, 0.066	<0.001, 0.182	0.306, 0.032	0.019, 0.082	<0.001, 0.141	0.409, 0.029	<0.001, 0.169	0.046, 0.064	0.071, 0.058	0.002, 0.098	<0.001, 0.121	0.293, 0.034	<0.001, 0.153
Willing to follow	0.118, 0.052	0.230, 0.045	0.161, 0.047	0.061, 0.064	0.003, 0.098	0.734, 0.011	0.008, 0.092	0.867, 0.005	<0.001, 0.114	<0.001, 0.146	0.193, 0.041	0.012, 0.088	0.613, 0.018	0.135, 0.053	0.121, 0.055	0.001, 0.109	<0.001, 0.142	0.259, 0.036	0.285, 0.035	0.022, 0.075	0.001, 0.108
Support promotional campaigns	0.538, 0.020	0.041, 0.077	0.608, 0.017	0.138, 0.051	<0.001, 0.119	0.192, 0.044	0.008, 0.092	0.229, 0.038	0.028, 0.070	<0.001, 0.136	0.450, 0.024	0.095, 0.058	0.449, 0.027	0.252, 0.040	0.001, 0.115	0.301, 0.033	0.002, 0.100	0.054, 0.061	0.036, 0.068	0.004, 0.094	<0.001, 0.159
Textbooks	<0.001, 0.237	0.940, 0.003	0.365, 0.030	0.816, 0.008	0.198, 0.043	<0.001, 0.154	<0.001, 0.233	<0.001, 0.213	<0.001, 0.233	<0.001, 0.200	0.005, 0.088	0.356, 0.032	0.768, 0.011	0.201, 0.045	<0.001, 0.153	0.486, 0.023	0.679, 0.013	<0.001, 0.126	0.273, 0.036	<0.001, 0.148	<0.001, 0.113
Campus promotion	0.020, 0.077	0.398, 0.032	0.574, 0.019	0.492, 0.023	0.290, 0.036	0.631, 0.016	0.002, 0.106	0.002, 0.096	0.002, 0.101	0.186, 0.042	0.052, 0.061	0.719, 0.013	0.821, 0.008	0.062, 0.066	0.184, 0.047	0.885, 0.005	0.086, 0.055	0.270, 0.035	0.069, 0.059	0.222, 0.040	0.543, 0.020
Lectures	<0.001, 0.152	0.459, 0.028	0.007, 0.090	0.956, 0.002	0.088, 0.057	0.073, 0.060	<0.001, 0.174	<0.001, 0.241	<0.001, 0.155	<0.001, 0.209	<0.001, 0.212	0.082, 0.061	0.477, 0.026	0.410, 0.029	0.365, 0.032	0.006, 0.088	0.309, 0.032	0.355, 0.029	0.087, 0.056	0.072, 0.059	0.926, 0.003
Broadcasting	<0.001, 0.196	0.153, 0.053	0.374, 0.030	0.933, 0.003	0.629, 0.016	0.505, 0.022	<0.001, 0.239	<0.001, 0.227	<0.001, 0.195	<0.001, 0.182	<0.001, 0.176	0.501, 0.024	0.122, 0.056	0.054, 0.068	0.036, 0.074	0.312, 0.033	0.148, 0.046	0.100, 0.052	0.680, 0.013	0.546, 0.020	0.846, 0.006
Magazine	<0.001, 0.251	0.199, 0.048	0.737, 0.011	0.024, 0.077	0.001, 0.110	<0.001, 0.169	<0.001, 0.201	<0.001, 0.246	<0.001, 0.181	<0.001, 0.148	0.005, 0.088	0.301, 0.036	0.423, 0.029	0.078, 0.062	0.001, 0.120	0.040, 0.066	0.002, 0.101	<0.001, 0.160	0.286, 0.035	0.004, 0.095	0.060, 0.060
Network	<0.001, 0.173	0.068, 0.068	0.952, 0.002	0.122, 0.053	0.038, 0.070	<0.001, 0.190	0.003, 0.104	<0.001, 0.177	<0.001, 0.123	0.001, 0.106	0.598, 0.017	0.048, 0.069	0.675, 0.015	0.951, 0.002	0.001, 0.121	0.088, 0.055	0.023, 0.073	<0.001, 0.194	0.590, 0.018	<0.001, 0.140	<0.001, 0.122
Family	<0.001, 0.296	0.361, 0.034	0.147, 0.048	0.282, 0.037	0.002, 0.105	0.001, 0.110	<0.001, 0.258	<0.001, 0.260	<0.001, 0.269	<0.001, 0.205	<0.001, 0.201	0.679, 0.014	0.939, 0.003	0.046, 0.071	0.033, 0.075	0.708, 0.012	0.066, 0.058	0.047, 0.063	0.031, 0.070	0.021, 0.075	0.883, 0.005
Peers	<0.001, 0.289	0.523, 0.024	0.002, 0.101	0.187, 0.045	0.001, 0.116	<0.001, 0.142	<0.001, 0.259	<0.001, 0.201	<0.001, 0.254	<0.001, 0.185	<0.001, 0.160	0.029, 0.076	0.560, 0.021	0.010, 0.091	0.001, 0.116	0.072, 0.058	0.631, 0.015	0.189, 0.042	0.024, 0.073	0.002, 0.100	0.408, 0.027
Others	0.107, 0.053	0.206, 0.047	0.845, 0.007	0.142, 0.050	0.451, 0.025	0.564, 0.019	_	0.078, 0.055	0.005, 0.090	0.302, 0.033	0.039, 0.065	0.167, 0.048	0.027, 0.079	0.419, 0.029	0.105, 0.057	0.158, 0.046	0.331, 0.031	0.677, 0.013	0.027, 0.072	0.313, 0.033	0.374, 0.043

* E represents East China; S represents South China; N represents North China; C represents Central China; SW represents Southwest China; NW represents Northwest China; NE represents Northeast China. *p* < 0.05 indicates that the behavior adoption rates among different groups are not completely the same, and there are statistically significant differences.

**Table 6:** The formation rate of water intake behaviors among the survey subjects.

Demographic Indicators		Water Intake Time										Correct Water Intake Pattern			
		Morning Empty Stomach	After Naps	Before Sleeping	After Strenuous Exercise	After Bathing	Before Meals	During Meals	After Meals	Feeling Thirsty	When Remember	Drink Water Regularly and in Moderation	Drink a Small Amount of Water When Thirsty	Drink Water Regularly and in Moderation	Others
Sex	Male	56.0% (53.4–58.5)	40.6% (38.1–43.2)	43.3% (40.7–45.9)	68.9% (66.5–71.2)	31.3% (28.9–33.7)	25.7% (23.4–28.1)	44.2% (41.7–46.8)	46.4% (43.8–49.0)	75.6% (73.3–77.8)	56.5% (53.9–59.1)	22.9% (20.8–25.2)	48.7% (46.1–51.3)	27.3% (25.0–29.7)	1.1% (0.6–1.7)
	Female	56.1% (53.7–58.4)	43.3% (41.0–45.7)	36.1% (33.9–38.4)	69.3% (67.0–71.5)	37.1% (34.8–39.4)	28.2% (26.1–30.4)	45.2% (42.8–47.6)	48.2% (45.8–50.6)	79.2% (77.2–81.1)	59.5% (57.2–61.8)	19.3% (17.5–21.2)	51.2% (48.8–53.6)	27.6% (25.5–29.8)	1.9% (1.3–2.6)
*p*		0.970	0.095	<0.001 *	0.802	<0.001 *	0.088	0.554	0.276	0.007 *	0.074	0.002			
**Effect size**		−0.001	−0.030	0.088	−0.004	−0.070	−0.030	−0.010	−0.019	−0.048	−0.032	0.068			
Area *	E	59.0% (54.1–63.8)	36.9% (32.3–41.8)	34.4% (29.9–39.2)	76.1% (71.7–80.1)	36.2% (31.6–41.0)	17.6% (14.2–21.6)	34.4% (29.9–39.2)	39.9% (35.3–44.8)	82.2% (78.1–85.6)	65.8% (61.0–70.3)	12.6% (9.7–16.2)	56.5% (51.6–61.3)	27.6% (23.5–32.2)	3.3% (1.9–5.5)
	S	49.5% (45.2–53.8)	42.4% (38.2–46.7)	47.4% (43.1–51.7)	57.1% (52.8–61.3)	43.5% (39.3–47.8)	40.6% (36.5–44.9)	44.5% (40.3–48.8)	52.6% (48.3–56.9)	61.3% (57.0–65.4)	52.4% (48.1–56.7)	28.4% (24.7–32.5)	39.7% (35.5–43.9)	31.1% (27.3–35.3)	0.8% (0.3–2.0)
	N	54.6% (49.1–60.0)	40.7% (35.4–46.2)	36.3% (31.2–41.7)	74.8% (69.7–79.2)	34.7% (29.7–40.1)	20.2% (16.1–25.0)	41.6% (36.3–47.1)	50.5% (45.0–55.9)	83.3% (78.8–87.0)	60.3% (54.8–65.5)	20.2% (16.1–25.0)	49.2% (43.8–54.7)	28.4% (23.7–33.6)	2.2% (1.1–4.5)
	C	57.0% (52.6–61.3)	42.4% (38.1–46.8)	34.6% (30.6–38.9)	75.6% (71.6–79.2)	29.4% (25.6–33.5)	17.2% (14.1–20.8)	41.2% (37.0–45.6)	44.8% (40.5–49.2)	87.8% (84.6–90.4)	62.0% (57.7–66.1)	22.4% (19.0–26.3)	54.6% (50.2–58.9)	20.4% (17.1–24.2)	2.6% (1.5–4.4)
	SW	53.3% (48.7–57.8)	41.0% (36.6–45.6)	35.4% (31.1–39.9)	67.0% (62.6–71.2)	21.6% (18.1–25.6)	24.9% (21.2–29.0)	42.6% (38.1–47.1)	45.2% (40.7–49.8)	77.9% (73.9–81.5)	54.8% (50.2–59.3)	15.9% (12.9–19.6)	55.5% (50.9–59.9)	27.7% (23.8–32.0)	0.9% (0.3–2.2)
	NW	59.6% (55.2–63.9)	44.3% (40.0–48.8)	39.4% (35.1–43.8)	71.1% (66.9–75.0)	37.9% (33.7–42.3)	29.1% (25.2–33.3)	52.2% (47.7–56.6)	49.7% (45.3–54.1)	83.3% (79.7–86.4)	61.4% (57.0–65.7)	18.1% (15.0–21.8)	47.4% (43.0–51.9)	33.8% (29.7–38.1)	0.6% (0.2–1.8)
	NE	59.5% (55.0–63.7)	45.7% (41.3–50.1)	45.5% (41.1–49.9)	65.6% (61.3–69.7)	37.4% (33.3–41.8)	35.2% (31.1–39.5)	53.9% (49.5–58.3)	48.6% (44.1–53.0)	70.8% (66.6–74.6)	52.7% (48.2–57.1)	26.1% (22.4–30.2)	49.4% (45.0–53.8)	23.7% (20.1–27.6)	0.8% (0.3–2.1)
*p*		0.009 **	0.211	<0.001 **	<0.001 **	<0.001 **	<0.001 **	<0.001 **	0.004 **	<0.001 **	<0.001 **	<0.001 **			
**Effect size**		0.073	0.052	0.105	0.142	0.141	0.192	0.125	0.078	0.212	0.098	0.358			

* E represents East China; S represents South China; N represents North China; C represents Central China; SW represents Southwest China; NW represents Northwest China; NE represents Northeast China. ** *p* < 0.05 indicates that the attitude endorsement rates among different groups are not completely the same, and there are statistically significant differences.

**Table 7:** Pairwise comparison of the adoption rates of water intake behaviors among the survey subjects in different regions (*p*-value and Cramér’s V).

AREA		E						S					N				C			SW		NW
AREA *		S	N	C	SW	NW	NE	N	C	SW	NW	NE	C	SW	NW	NE	SW	NW	NE	NW	NE	NE
Water intake time	Waking up on an empty stomach in the morning	0.004, 0.095	0.230, 0.045	0.537, 0.021	0.090, 0.058	0.870, 0.005	0.899, 0.004	0.156, 0.049	0.017, 0.075	0.241, 0.038	0.001, 0.101	0.002, 0.100	0.496, 0.024	0.721, 0.013	0.160, 0.050	0.171, 0.048	0.247, 0.037	0.410, 0.026	0.433, 0.025	0.051, 0.064	0.055, 0.062	0.969, 0.001
	After taking a nap	0.097, 0.055	0.305, 0.038	0.097, 0.055	0.219, 0.042	0.026, 0.075	0.009, 0.088	0.636, 0.016	0.990, 0.000	0.678, 0.013	0.529, 0.020	0.290, 0.033	0.630, 0.017	0.921, 0.004	0.309, 0.036	0.164, 0.049	0.672, 0.014	0.541, 0.019	0.300, 0.033	0.309, 0.033	0.151, 0.047	0.673, 0.014
	Before sleep	<0.001, 0.130	0.606, 0.019	0.956, 0.002	0.771, 0.010	0.129, 0.051	0.001, 0.112	0.002, 0.109	<0.001, 0.130	<0.001, 0.122	0.011, 0.081	0.543, 0.019	0.625, 0.017	0.796, 0.009	0.376, 0.031	0.010, 0.091	0.803, 0.008	0.120, 0.050	<0.001, 0.111	0.203, 0.041	0.002, 0.103	0.055, 0.062
	After strenuous exercise	<0.001, 0.199	0.673, 0.016	0.854, 0.006	0.003, 0.100	0.095, 0.056	0.001, 0.114	<0.001, 0.179	<0.001, 0.196	0.001, 0.102	<0.001, 0.146	0.005, 0.088	0.787, 0.009	0.021, 0.083	0.260, 0.040	0.006, 0.097	0.003, 0.095	0.113, 0.051	0.001, 0.109	0.173, 0.044	0.651, 0.015	0.066, 0.059
	After a shower	0.025, 0.074	0.681, 0.015	0.031, 0.072	<0.001, 0.161	0.591, 0.018	0.698, 0.013	0.012, 0.087	<0.001, 0.147	<0.001, 0.232	0.072, 0.057	0.050, 0.062	0.112, 0.056	<0.001, 0.145	0.352, 0.033	0.429, 0.028	0.006, 0.089	0.005, 0.090	0.007, 0.085	<0.001, 0.178	<0.001, 0.173	0.875, 0.005
	Before meals	<0.001, 0.248	0.376, 0.033	0.879, 0.005	0.009, 0.089	<0.001, 0.134	<0.001, 0.197	<0.001, 0.211	<0.001, 0.258	<0.001, 0.167	<0.001, 0.121	0.076, 0.056	0.282, 0.038	0.126, 0.055	0.005, 0.100	<0.001, 0.161	0.003, 0.095	<0.001, 0.141	<0.001, 0.205	0.149, 0.047	0.001, 0.112	0.041, 0.065
	During meals	0.002, 0.102	0.048, 0.074	0.038, 0.069	0.015, 0.083	<0.001, 0.178	<0.001, 0.195	0.421, 0.028	0.290, 0.033	0.548, 0.019	0.015, 0.077	0.003, 0.094	0.901, 0.004	0.795, 0.009	0.004, 0.103	0.001, 0.120	0.666, 0.014	0.001, 0.110	<0.001, 0.127	0.003, 0.096	<0.001, 0.113	0.586, 0.017
	After meals	<0.001, 0.126	0.005, 0.105	0.144, 0.049	0.122, 0.053	0.004, 0.097	0.010, 0.086	0.549, 0.021	0.013, 0.078	0.021, 0.074	0.355, 0.029	0.200, 0.040	0.113, 0.055	0.148, 0.052	0.828, 0.008	0.596, 0.019	0.902, 0.004	0.124, 0.049	0.237, 0.038	0.167, 0.045	0.301, 0.034	0.724, 0.011
	Feeling thirsty	<0.001, 0.226	0.694, 0.015	0.018, 0.079	0.125, 0.052	0.656, 0.015	<0.001, 0.132	<0.001, 0.232	<0.001, 0.303	<0.001, 0.180	<0.001, 0.245	0.002, 0.100	0.069, 0.064	0.067, 0.066	0.995, 0.000	<0.001, 0.142	<0.001, 0.131	0.044, 0.064	<0.001, 0.210	0.037, 0.068	0.012, 0.082	<0.001, 0.149
	When remember	<0.001, 0.135	0.124, 0.057	0.236, 0.040	0.001, 0.112	0.178, 0.045	<0.001, 0.133	0.027, 0.077	0.002, 0.097	0.456, 0.024	0.004, 0.091	0.935, 0.003	0.617, 0.017	0.132, 0.054	0.735, 0.012	0.035, 0.075	0.024, 0.073	0.857, 0.006	0.003, 0.094	0.039, 0.067	0.512, 0.021	0.006, 0.089
Correct water intake pattern		<0.001, 0.423	0.870, 0.006	0.221, 0.041	<0.001, 0.192	0.119, 0.053	<0.001, 0.268	<0.001, 0.411	<0.001, 0.404	<0.001, 0.262	<0.001, 0.393	<0.001, 0.181	0.192, 0.046	<0.001, 0.191	0.107, 0.057	<0.001, 0.262	<0.001, 0.157	0.710, 0.012	<0.001, 0.238	<0.001, 0.146	0.009, 0.085	<0.001, 0.227

* E represents East China; S represents South China; N represents North China; C represents Central China; SW represents Southwest China; NW represents Northwest China; NE represents Northeast China. *p* < 0.002, which is the level of one-sided test, indicates that when making pairwise comparisons among different geographical intervals, there are statistically significant differences in the behavior adoption rate.

## Data Availability

The original contributions presented in this study are included in the article. Further inquiries can be directed to the corresponding author.

## Abbreviations

The following abbreviations are used in this manuscript:

E	East China
S	South China
N	North China
C	Central China
SW	Southwest China
NW	Northwest China
NE	Northeast China
